# Return‐to‐sport and performance outcomes after Achilles tendon rupture in National Hockey League players: A matched cohort study

**DOI:** 10.1002/ksa.70458

**Published:** 2026-05-22

**Authors:** David Slawaska‐Eng, Harjind Kahlon, Marc Daniel Bouchard, John Theodoropoulos, Alexander E. Weber, Olufemi Ayeni

**Affiliations:** ^1^ Department of Orthopaedic Surgery, Keck School of Medicine University of Southern California Los Angeles California USA; ^2^ Temerty Faculty of Medicine University of Toronto Toronto Ontario Canada; ^3^ Division of Orthopaedic Surgery, Department of Surgery McMaster University Hamilton Ontario Canada; ^4^ Division of Orthopaedic Surgery, Department of Surgery University of Toronto Orthopaedic Sports Medicine Program (UTOSM) Toronto Ontario Canada

**Keywords:** Achilles tendon rupture, ice hockey, National Hockey League, performance outcomes, return to sport

## Abstract

**Purpose:**

Achilles tendon ruptures (ATRs) are uncommon but career‐threatening injuries in elite athletes. Limited evidence exists regarding return‐to‐sport (RTS) and performance outcomes among National Hockey League (NHL) players following ATR. This study aimed to evaluate RTS rates and post‐injury performance metrics in NHL players after ATR, using matched controls for comparison.

**Methods:**

NHL players who sustained an ATR between the 2000–2001 and 2024–2025 seasons were identified using publicly available databases and confirmed through independent sports news sources. Each player was matched 1:1 to a control based on position, age and pre‐injury performance using a validated similarity algorithm. Demographic data and performance metrics, including points per game (PPG), games played (GP), average time on ice (ATOI) and a validated hockey‐specific performance score (PS), were collected for two seasons before and after the injury or index date. RTS rates and pre‐ versus post‐injury changes were assessed using paired *t* tests, with significance set at *p* < 0.05.

**Results:**

A total of 20 NHL players met the inclusion criteria and were successfully matched to controls. The overall RTS rate was 87.0%, rising to 100% when accounting for players who retired from the NHL but continued playing professionally in other leagues. Although players with ATR (cases) demonstrated performance declines post‐injury, no significant differences were observed in PS, PPG, ATOI or GP at any position, except for PS when all positions were analysed collectively. Similarly, control players experienced non‐significant performance declines across all metrics post‐index.

**Conclusion:**

ATR in NHL players is associated with high RTS rates and largely preserved performance metrics. While a modest decline in PS was observed, other key metrics remained stable compared to matched controls. These findings support favourable functional recovery and can inform post‐injury expectations and management.

**Level of Evidence:**

Level III.

**Abbreviations**
ATOI: average time on ice
ATR: Achilles tendon rupture
CI: confidence interval
ESG: even‐strength goals
GP: games played
GWG: game‐winning goals
NHL: National Hockey League
PIM: penalties in minutes
PPG: points per game
PS: performance score
PWPG: power play goals
RTS: return to sport

## INTRODUCTION

Ice hockey is a high‐intensity sport that demands a combination of speed, agility and power, placing substantial biomechanical stress on the lower extremities [22]. The sport's dynamic nature, characterized by rapid accelerations, sudden decelerations and frequent directional changes, predisposes athletes to a variety of musculoskeletal injuries [2, 26]. While hip and groin injuries have been extensively studied in this population, less attention has been given to Achilles tendon ruptures (ATRs) [23]. This likely stems from the relative rarity of ATRs in ice hockey, as the skating stride involves reduced eccentric loading of the Achilles complex compared to other high‐impact sports [20].

The Achilles tendon is the strongest and thickest tendon in the human body and plays a critical role in force generation during plantarflexion, contributing to propulsion during skating despite relatively lower eccentric loading demands [18]. Rupture of this tendon is a severe injury that can lead to prolonged rehabilitation and, in some cases, diminished athletic performance [16]. In the general population, the incidence of ATRs is estimated at approximately between 8 and 18 per 100,000 persons, with higher rates observed among athletes engaged in sports requiring explosive lower limb movements [3, 16].

In professional sports, ATRs have been associated with variable return‐to‐sport (RTS) rates and performance outcomes [13]. For instance, studies have reported RTS rates ranging from 61% to 100% among elite male athletes following surgical repair of ATRs [13]. However, the impact on post‐injury performance varies across sports, with some athletes experiencing significant declines in strength and function for up to 2–3 years post‐injury, and others never fully restoring pre‐injury performance [13, 16]. Specifically, in the National Basketball Association (NBA), players have demonstrated decreased performance metrics post‐ATR [14].

Within the context of the National Hockey League (NHL), data on ATRs remain limited. A recent study identified 15 NHL players who sustained ATRs between 2001 and 2021, reporting a high return to play rate of 93% and no significant differences in performance metrics compared with controls [7]. Their work provided a critical foundation, but given the rarity of ATRs in hockey, further research is needed to confirm and extend these findings.

The purpose of this study is to evaluate the RTS rate and performance‐based outcomes among NHL players following ATR through the 2024–2025 season. While ATR has been extensively studied in other sports, limited sport‐specific research for ice hockey exists. By utilizing a matched control design and standardized performance metrics, this study aims to provide a comprehensive assessment of the impact of ATRs on professional ice hockey players. We hypothesize that while the RTS rate will be high, players may experience a decline in performance compared to matched controls, highlighting the need for targeted rehabilitation strategies and informed clinical decision‐making.

## METHODS

### Player identification

By using previously published methodologies, NHL players who sustained ATRs between the 2000–2001 and 2024–2025 seasons were identified from the publicly available NHL Injury Viz database [6, 15, 17, 21, 23, 25]. To ensure completeness and account for potential misclassification of Achilles' injuries, all players with injuries classified as ‘Achilles’, ‘leg’, ‘lower body’. ‘Ankle’, ‘foot’, ‘heel’ and ‘calf’ were extracted. Players who sustained ATRs were confirmed through publicly available sports reporting websites, including TSN.ca, ESPN and FoxSports.com.

Players were included in the study if they were placed on the injured list and had their injury and treatment corroborated by at least two independent public sources. Due to limitations of publicly available data, definitive confirmation of operative versus non‐operative management was not possible in all cases. To minimize confounding variables, players with concomitant injuries requiring surgery were excluded.

### Matched control selection

A validated similarity algorithm based on position, age and performance was used to identify 1:1 matched controls [21]. Controls were required to have played during the same era as their matched case and not sustained any major lower‐extremity injury. On the basis of matched cases' years of NHL experience at the time of injury, each control player was assigned an index date to compare performance pre‐injury/pre‐index and post‐injury/post‐index.

### Demographic and injury variables

Data collected for each player included age at time of injury, height, weight, position, games missed, mechanism of injury, laterality of injury (R vs. L) and years of NHL experience at the time of injury.

### Data abstraction

For eligible players, all regular‐season game statistics were retrieved using Hockey‐Reference.com, covering data from their rookie year through the 2024–2025 seasons [8]. To ensure data reliability, a sample extraction of ten players' statistics was conducted by two independent reviewers and verified by a senior author before proceeding with full data extraction.

Variables extracted for forwards and defensemen included games played (GP) per season, goals, assists, points, plus/minus rating, penalties in minutes (PIM), even‐strength goals (ESG), power play goals (PWPG), game‐winning goals (GWG), shots on goal (SOG) and average time on ice (ATOI). Pre‐ and post‐operative statistics were obtained. The post‐operative period was defined by the first season following surgery.

### Outcome measures

Performance‐based outcomes included the RTS rate, defined as returning to the active roster for at least one professional regular‐season NHL game. RTS to other professional leagues was not included in this definition in order to confine the analysis to the single highest level of competition. Additional metrics included GP per season, points per game (PPG), ATOI and a validated hockey‐specific performance score (PS) as described by Schroeder et al. (Table 1) [21]. Pre‐ and post‐ATR PS were analysed, stratified by position into forward, defensemen and all players combined. Cases and matched controls were assessed for baseline equivalency in terms of PS and career statistics, including PPG, ATOI and GP per season.

**Table 1:** Performance score formula for a given position.

Forward	$\frac{\left(3.0\times \mathrm{ESG}\right)+\left(2.0\times \mathrm{PWPG}\right)+\left(4.0\times \mathrm{SHG}\right)+\left(4.0\times \;\mathrm{GWG}\right)+(2.0\times \mathrm{assists})+(\mathrm{plus}/\mathrm{minus})-(0.25\times \mathrm{PIM})-\left(0.33\times \mathrm{SOG}\right)}{\mathrm{Games}\;\mathrm{played}}$
Defenseman	$\frac{\left(5.0\times \mathrm{ESG}\right)+\left(4.0\times \mathrm{PWPG}\right)+\left(6.0\times \mathrm{SHG}\right)+\left(5.0\times \mathrm{GWG}\right)+(3.0\times \mathrm{assists})+(\mathrm{plus}/\mathrm{minus})-(0.25\times \mathrm{PIM})-\left(0.33\times \mathrm{SOG}\right)}{\mathrm{Games}\;\mathrm{played}}$
Goalie	$\frac{(0.7\times \mathrm{wins})+(0.2\times \mathrm{ties}\;\mathrm{plus}\;\mathrm{overtime}\;\mathrm{losses})+\mathrm{shutouts}+(0.17\times \mathrm{saves})-(0.25\times \mathrm{losses})-\left(1.23\times \mathrm{goals}\;\mathrm{against}\right)}{\mathrm{Games}\;\mathrm{played}}$

Abbreviations: ESG, even‐strength goals; GWG, game‐winning goals; PIM, penalty in minutes; PWPG, power play goals; SHG, short‐handed strength goals; SOG, shots on goal.

### Statistical analysis

Pre‐ and post‐operative statistics were calculated using Microsoft Excel version 16.90, with post‐operative performance defined by the sum of the season statistics beginning from the first full season following surgery. Continuous pre‐ and post‐operative variables were compared within each cohort and between cohorts and controls using paired *t* tests. Two‐proportion *z* test or *χ* ^2^ test was used for dichotomous variables. A post hoc power analysis was performed on all statistically significant outcomes, where high post hoc power is defined as >0.8. For all analyses, statistical significance was set at *p* < 0.05. All statistical analyses were performed using Excel version 16.90 (Microsoft Inc).

## RESULTS

### Study sample

**Figure 1:**
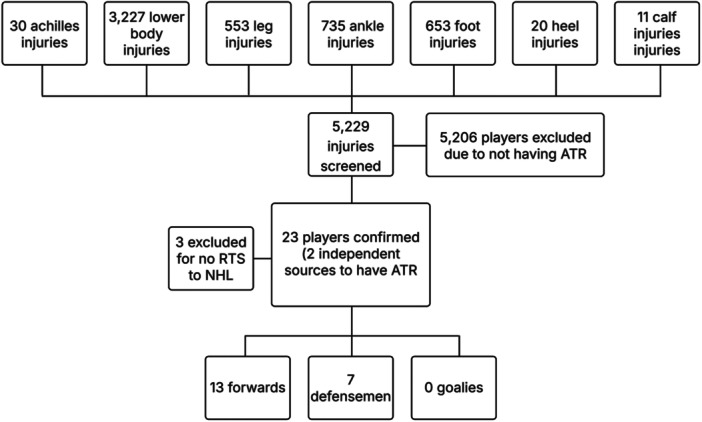
Flowchart of participants. ATR, Achilles tendon rupture; NHL, National Hockey League; RTS, return to sport.

A total of 5279 NHL player injuries (30 Achilles injuries, 3277 lower body injuries, 553 leg injuries, 735 ankle injuries, 653 foot injuries, 20 heel injuries and 11 calf injuries) between the 2000 and 2025 NHL seasons were initially screened to identify pertinent ATRs. In addition, 5260 players were excluded due to injuries unrelated to the Achilles tendon, and a further single player was excluded due to having a non‐rupture Achilles tendon injury. Three additional players were excluded due to not returning to play after ATR. The final sample consisted of 20 players (13 forwards and 7 defensemen) who underwent surgery for unilateral ATRs, with a mean age of 27.7 ± 4.8 years (range: 21–38) at the time of diagnosis (Table 2, Figure 1). Cases had a mean 8.0 ± 4.1 years of experience in the NHL prior to ATR and missed 40.2 ± 15.4 games.

**Table 2:** Player demographics.

	Cases					Control			
	Age (years)	Height (cm)	Weight (kg)	NHL experience at time of injury (years)	Games missed	Age (years)	Height (cm)	Weight (kg)	NHL experience at time of injury of case player (years)
Forwards	26	180	89	8	60	25	185	87	8
	34	185	98	14	21	32	188	93	14
	23	185	91	5	51	21	188	91	4
	29	178	86	11	43	31	180	91	11
	38	191	98	15	32	36	183	90	15
	23	183	87	5	36	26	190	94	5
	33	188	93	15	37	32	185	99	12
	25	175	78	7	42	27	180	86	6
	24	185	91	6	10	23	190	93	6
	31	183	93	9	51	32	188	102	9
	26	185	83	8	25	28	183	87	8
	21	185	83	2	32	21	183	83	2
	26	191	93	5	67	25	183	85	5
Defensemen	22	183	86	4	31	23	201	111	5
	30	183	88	5	56	26	190	99	4
	24	191	101	3	51	21	180	76	2
	26	188	101	7	19	27	180	92	7
	25	188	95	5	30	28	185	85	5
	35	191	96	12	52	30	183	90	11
	32	183	93	13	58	32	180	84	12
Forwards									
AVG	27.6	184.2	89.5	8.5	39.0	27.6	185.1	90.8	8.1
SD	5.0	4.6	5.9	4.2	15.8	4.7	3.5	5.5	4.0
Defensemen									
AVG	27.7	186.7	94.3	7.0	42.4	26.7	185.6	91.0	6.6
SD	4.7	3.7	5.8	4.0	15.4	3.8	7.7	11.4	3.7
All players									
AVG	27.7	185.1	91.2	8.0	40.2	27.3	185.3	90.9	7.6
SD	4.8	4.4	6.2	4.1	15.4	4.3	5.1	7.7	3.8

Abbreviations: AVG, average; NHL, National Hockey League; SD, standard deviation.

Matched control NHL players were selected based on the aforementioned algorithm. These 20 control players consisted of 13 forwards and 7 defensemen with no history of Achilles tendon injury and a mean age of 27.3 ± 4.3 years (range: 21–36) at the index date. Control players had a mean 7.6 ± 3.8 years of experience at this time point.

### RTS

The overall RTS rate for NHL players following ATR was 87.0% (20 of 23 players). Of the 20 players who returned to sport, 13 were forwards and 7 were defensemen. All three players who did not return to play in the NHL (one forward, one defenseman and one goalie) were no longer signed to their teams and went on to play professionally in other international leagues.

### Baseline equivalency

No statistically significant differences were found between cases and controls in pre‐ATR/pre‐index PS, PPG, GP per season or ATOI for all players combined, forwards or defensemen (*p* > 0.05).

### PSs

**Figure 2:**
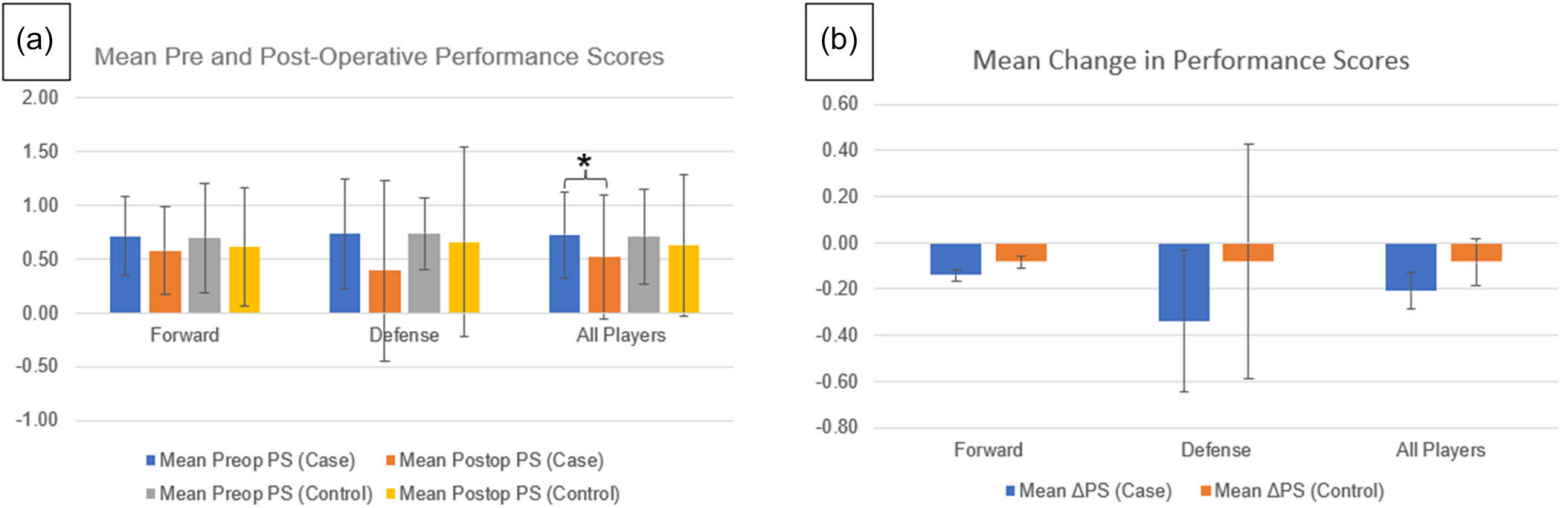
(a) Mean pre‐ and post‐operative Performance Scores (PSs) in forwards (*n* = 13), defensemen (*n* = 7), and all players pooled together (*n* = 20) for cases and controls. Error bars represent the standard deviation (SD). (b) Change in mean PS in forwards, defensemen and all players pooled for cases and controls. Error bars represent the 95% confidence interval (95% CI). The * indicates statistical significance (*p* < 0.05).

Declines in PS were observed in all groups, with a statistically significant decline for all players combined (pre‐ATR PS: 0.72  ±  0.40; post‐ATR PS: 0.51 ± 0.58; ∆PS = −0.21, 95% CI [−0.29, −0.13]; *p* = 0.035). No statistically significant changes were observed for forwards (pre‐ATR PS: 0.71 ± 0.36; post‐ATR PS: 0.57 ± 0.40; ∆PS = −0.14, 95% CI [−0.16, −0.11]; *p* = 0.247) or defensemen (pre‐ATR PS: 0.73 ± 0.51; post‐ATR PS: 0.39 ±  0.84; ∆PS = −0.34, 95% CI [−0.64, −0.03]; *p *= 0.072) (Table 3, Figure 2a).

**Table 3:** PS analysis.

		Cases				Control			
Position	*n*	Mean preop PS (±SD)	Mean postop PS (±SD)	Mean ∆PS [95% CI]	*p* value	Mean preop PS (±SD)	Mean postop PS (±SD)	Mean ∆PS [95% CI]	*p* value
Forward	13	0.71 ± 0.36	0.57 ± 0.40	−0.14 [−0.16, −0.11]	0.247	0.69 ± 0.50	0.61 ± 0.54	−0.08 [−0.11, −0.06]	0.591
Defense	7	0.73 ± 0.51	0.39 ± 0.84	−0.34 [−0.64, −0.03]	0.072	0.74 ± 0.33	0.65 ± 0.88	−0.08 [−0.59, 0.43]	0.831
All players	20	0.72 ± 0.40	0.51 ± 0.58	−0.21 [−0.29, −0.13]	0.035^a^	0.71 ± 0.44	0.63 ± 0.66	−0.08 [−0.18, 0.02]	0.600

Abbreviations: CI, confidence interval; PS, performance score; SD, standard deviation.

a Statistical significance (*p* < 0.05).

Pre‐ and post‐index PS were analysed for controls, showing declines in PS in all groups. No statistically significant changes were observed for all players combined, forwards or defensemen (Table 3, Figure 2a).

Comparison of pre‐injury/index PS and post‐injury/index PS showed no statistically significant differences between cases and controls for all players combined (*p* = 0.319), forwards (*p* = 0.640) or defensemen (*p* = 0.407) (Figure 2b).

### Career statistics

Comparison of cases' career statistics pre‐injury/index and post‐injury/index demonstrated no statistically significant differences with respect to PPG, GP per season or ATOI for all player positions.

There was a relatively consistent reduction in PPG for cases pre‐injury to post‐injury for all players combined, forwards and defensemen. Controls demonstrated a similar downward scoring trend for all three groups. Scoring was not significantly different between cases and controls at any position (*p* > 0.05) (Table 4).

**Table 4:** PPG analysis.

		Cases				Control			
Position	*n*	Mean preop PPG (±SD)	Mean postop PPG (±SD)	Mean ∆PPG [95% CI]	*p* value	Mean preop PPG (±SD)	Mean postop PPG (±SD)	Mean ∆PPG [95% CI]	*p* value
Forward	13	0.60 ± 0.20	0.56 ± 0.24	−0.04 [−0.16, 0.07]	0.414	0.59 ± 0.27	0.55 ± 0.26	−0.04 [−0.17, 0.08]	0.562
Defense	7	0.39 ± 0.20	0.31 ± 0.26	−0.07 [−0.17, 0.02]	0.116	0.39 ± 0.11	0.38 ± 0.27	−0.01 [−0.22, 0.21]	0.934
All players	20	0.53 ± 0.22	0.47 ± 0.27	−0.05 [−0.13, 0.02]	0.145	0.52 ± 0.25	0.49 ± 0.27	−0.03 [−0.14, 0.08]	0.584

Abbreviations: CI, confidence interval; PPG, points per game; SD, standard deviation.

While not statistically significant, there was a reduction in GP per season following ATR, particularly in defensemen (pre: 55.98 ± 13.93; post: 41.79 ± 22.46; ΔGP = −14.19, 95% CI [−32.98, 4.60]; *p *= 0.114). All positions combined and the forwards group also saw a slight decrease in GP per season. Control players had modest increases in GP per person post‐index in all positions combined, forwards and defensemen. No statistically significant differences were observed between cases and controls at any position (*p* > 0.05) (Table 5).

**Table 5:** GP per season analysis.

		Cases				Control			
Position	*n*	Mean preop GP/season (±SD)	Mean postop GP/season (±SD)	Mean ∆GP/season [95% CI]	*p* value	Mean preop GP/season (±SD)	Mean postop GP/season (±SD)	Mean ∆GP/season [95% CI]	*p* value
Forward	13	59.30 ± 11.11	59.61 ± 9.91	−0.66 [−8.43, 7.11]	0.862	56.80 ± 16.17	64.96 ± 13.32	8.17 [−5.12, 21.46]	0.201
Defense	7	55.98 ± 13.93	41.79 ± 22.46	−14.19 [−32.98, 4.60]	0.114	47.85 ± 23.81	52.85 ± 25.40	4.99 [−17.88, 27.87]	0.612
All players	20	58.14 ± 11.91	53.37 ± 17.24	−4.76 [−12.65, 3.12]	0.161	53.67 ± 19.06	60.72 ± 18.74	7.06 [−2.64, 16.76]	0.183

Abbreviations: CI, confidence interval; GP, games played; SD, standard deviation.

ATOI remained stable for cases in all three analysed groups. ATOI had a similar pattern in controls for all groups. There were no statistically significant differences in ATOI between cases and controls at any position (*p *> 0.05) (Table 6).

**Table 6:** ATOI analysis.

		Cases				Control			
Position	*n*	Mean preop ATOI (±SD)	Mean postop ATOI (±SD)	Mean ∆ATOI [95% CI]	*p* value	Mean preop ATOI (±SD)	Mean postop ATOI (±SD)	Mean ∆ATOI [95% CI]	*p* value
Forward	13	16.12 ± 1.71	16.20 ± 2.14	−0.01 [−1.23, 1.20]	0.983	14.73 ± 4.47	15.56 ± 3.27	0.83 [−1.15, 2.81]	0.842
Defense	7	19.99 ± 2.69	19.04 ± 3.45	−0.96 [−3.14, 1.23]	0.325	19.37 ± 2.91	18.69 ± 5.87	−0.68 [−4.04, 2.68]	0.639
All players	20	17.48 ± 2.78	17.19 ± 2.93	−0.28 [−1.28, 0.71]	0.471	16.36 ± 4.52	16.66 ± 4.47	0.30 [−1.09, 1.69]	0.826

Abbreviations: ATOI, average time on ice; CI, confidence interval; SD, standard deviation.

## DISCUSSION

Despite the historically career‐altering perception of ATRs in professional sports, these findings suggest that NHL players experience high RTS rates and largely preserved performance following surgical repair. Our results are consistent with a prior NHL‐specific investigation by Hayes et al. [7], which also reported high RTS rates and stable post‐injury performance. In this study, 87.0% of players were able to return to play, and the remaining players who were not able to do so in the NHL continued to play professional hockey in other leagues, indicating substantial functional recovery. Additionally, player performance metrics such as PPG, ATOI and GP per season did not show any significant decline for cases' performance post‐injury. Although there was a statistically significant drop in PS for all players combined, position‐stratified analyses revealed no significant differences. Taken together, these findings may suggest that contemporary surgical repair and rehabilitation protocols facilitate reliable RTS in NHL players without major compromises in measurable on‐ice performance.

The RTS rate observed in this cohort (87%) is comparable to the 93% reported by Hayes et al. [7]. The modest discrepancy likely reflects differences in methodology and cohort composition rather than a true divergence in outcomes. Hayes et al. analysed 15 players, and the small sample size may have inflated the apparent RTS rate, whereas our broader screening strategy captured 20 players across a longer study window. By extending the study window and applying a more inclusive case‐identification strategy across multiple injury designations, our analysis aimed to maximize case capture in the setting of a rare injury rather than reflect differences in surgical management. Nevertheless, both studies converge on the conclusion that NHL athletes demonstrate high RTS following ATR, reinforcing the generalizability of these findings across analytic approaches.

It is important to note that RTS in this study was defined as return to NHL participation specifically, not to other leagues. This may underestimate true functional recovery following ATR, as several players who did not return to the NHL continued to play professionally in other leagues.

The importance of a matched‐control design in evaluating performance after injury has been highlighted previously, including by Sochacki et al. in their analysis of NHL players undergoing hip arthroscopy for femoroacetabular impingement syndrome [24]. Consistent with their observations, our study found that modest declines in PS, PPG, GP per season and ATOI among cases closely paralleled those in controls, supporting the interpretation that performance trajectories largely reflect aging and natural career progression rather than ATR‐specific effects. Furthermore, given the league's mean retirement age of 27.0 years and this cohort's average age of 27.7 years [4], it is plausible that age‐related decline, roster competition and contract dynamics contributed to these reductions. This may also explain why the three players who did not return to the NHL continued their careers in international professional leagues.

Interestingly, while other performance metrics saw modest decreases post‐ATR, ATOI remained stable, which may reflect continued trust in players' abilities from a coaching and organizational standpoint. In their analysis of performance following shoulder instability in the NHL, Swindell et al. observed an increase in ATOI relative to matched controls, postulating that coaching staff may be incentivized to give more playing time to newly returned players and provide reprieve to the rest of the team [25]. While increased ATOI is unlikely following major injury, its stability in our cohort suggests that teams reintegrate players at comparable workloads once cleared, indicating confidence in functional recovery rather than preferential deployment.

In other sports, RTS rates following ATR have ranged between 61%–79% in the NBA, 61%–72% in the NFL and 71%–78% in professional soccer leagues, with significant detriment to player performance universally [1, 5, 10, 11, 27, 28, 29]. Particularly in NBA players, Trofa et al. found that in their first season back following an ATR, they played significantly fewer games (67.5%), had fewer minutes (52.8%) and decreased player efficiency ratings [27]. These trends persisted into the second post‐ATR season. Lemme et al. further confirmed these trends, observing that one‐third of players did not return following ATR [14]. These differences likely reflect the sport‐specific biomechanics of tendon loading: basketball and football involve repeated jumping, sprinting and directional changes, which place high eccentric load on the Achilles tendon as the dorsiflexed foot resists motion prior to plantarflexion [13, 20]. This generates substantial tendon stress, increasing rupture risk and complicating recovery. By contrast, ice hockey involves primarily concentric propulsion and the gliding nature of skating may reduce cumulative tendon strain [20]. Additionally, the rigid structure of hockey skate boots may limit extremes of dorsiflexion and plantarflexion, further reducing stress on the tendon [20]. These biomechanical and equipment‐related differences plausibly contribute to the higher RTS rates and performance preservation observed among NHL players post‐ATR.

While no standardized guidelines exist for ATR management, all players in this cohort were treated operatively. Several studies have demonstrated that surgical management may offer superior functional outcomes, quicker RTS and lower re‐rupture rates, all of which may influence players' and teams' choice of management [12, 16, 19, 30]. However, outcomes of nonoperative management have improved with early functional rehabilitation, and in some populations may approach those of surgery [16, 19]. As evidence evolves, individualized decision‐making accounting for player role, contract context and organizational risk tolerance will be essential. Future prospective work comparing operative and nonoperative approaches in elite hockey players would help refine management strategies.

Strengths of this study include its focus on a rare but clinically important injury in elite hockey players, the largest NHL ATR cohort analysed to date (to the authors' knowledge) and the use of a matched‐control design to contextualize performance outcomes. By extending the study period through the 2024–2025 season and applying a rigorous screening methodology across multiple injury designations, we maximized case capture and minimized misclassification. Furthermore, the incorporation of validated hockey‐specific performance metrics (PS, PPG, GP and ATOI) provides a multifaceted evaluation of recovery that extends beyond single metrics used in prior work. Together, these features enhance both the internal validity and clinical applicability of the findings.

Despite these strengths, several limitations warrant consideration. Reliance on publicly available data introduces potential selection and reporting bias, as injury details are often underreported or intentionally obscured, particularly for less‐prominent players. We lacked access to surgical details, rehabilitation protocols and patient‐reported outcome measures, which would have provided a more nuanced understanding of recovery. The modest sample size (*n* = 20) limited statistical power, particularly in position‐stratified analyses; post hoc power analysis indicated only moderate power (0.44) to detect differences in PS, raising the possibility of Type II error. Post hoc power analysis is dependent on observed data and may not provide additional meaningful information beyond *p* values and CIs. Consequently, post hoc power analyses can be considered controversial and may be misleading when used to justify non‐significant findings [9]. In addition, while the matched‐control design strengthened internal comparisons, residual confounding from unmeasured factors such as coaching strategies, linemates or roster roles cannot be excluded. Future studies with larger cohorts, access to surgical and rehabilitation data and integration of advanced workload or tracking metrics would further refine prognostication and guide individualized return‐to‐play strategies.

## CONCLUSION

ATR does not appear to be career‐ending in NHL players. Most athletes successfully returned to play, and performance outcomes largely mirrored those of matched controls, suggesting that modest declines were more related to aging than to ATR‐specific deficits. Compared with other high‐impact sports, the biomechanics of hockey and skate support may mitigate long‐term consequences. Surgical repair and rehabilitation appear effective, though larger studies with surgical detail, rehabilitation data and player‐reported outcomes are needed to refine return‐to‐play expectations.

## AUTHOR CONTRIBUTIONS

David Slawaska‐Eng conceived and designed the study, performed the statistical analysis and drafted the manuscript. Marc Daniel Bouchard and Harjind Kahlon contributed to data collection and manuscript writing. John Theodoropoulos, Alexander E. Weber and Olufemi Ayeni contributed to manuscript editing and critical revisions. All authors reviewed and approved the final manuscript.

## ACKNOWLEDGEMENTS

The authors have no funding to report.

## CONFLICT OF INTEREST STATEMENT

Olufemi Ayeni receives speaker fees from Stryker Canada and research funding from the Canada Research Chair; however, none of the fees or funding were used to complete this study. Alexander E. Weber serves as Medical Director and Head Team Physician for the Los Angeles Kings and receives educational support from Stryker USA, which was not used to complete this study. John Theodoropoulos serves as Head Team Physician for the Toronto Maple Leafs. The remaining authors declare no conflicts of interest.

## ETHICS STATEMENT

Ethics approval was not required for this study as all data were obtained from publicly available sources and did not involve human participants directly or identifiable private information. Patient consent was not required as only publicly available information was used.

## Data Availability

The data analysed in this study are publicly available and can be accessed from publicly accessible sources. No new data were generated during this study. Analysed raw data from the current study are available from the corresponding author on reasonable request.

## References

[1] Amin NH , Old AB , Tabb LP , Garg R , Toossi N , Cerynik DL . Performance outcomes after repair of complete Achilles tendon ruptures in National Basketball Association players. Am J Sports Med. 2013;41(8):1864–1868. 23733634 10.1177/0363546513490659

[2] Anderson GR , Melugin HP , Stuart MJ . Epidemiology of injuries in ice hockey. Sports Health. 2019;11(6):514–519. 31158326 10.1177/1941738119849105 PMC6822215

[3] Bhandari M , Guyatt GH , Siddiqui F , Morrow F , Busse J , Leighton RK , et al. Treatment of acute Achilles tendon ruptures: a systematic overview and metaanalysis. Clin Orthop Relat Res. 2002;400:190–200. 10.1097/00003086-200207000-00024 12072762

[4] Cavan E , Cao J , Swartz TB . NHL aging curves using functional principal component analysis. J Quant Anal Sports. 2025;21(3):177–189.

[5] Forlenza EM , Lavoie‐Gagne OZ , Lu Y , Diaz CC , Chahla J , Forsythe B . Return to play and player performance after Achilles tendon rupture in UEFA professional soccer players: a matched‐cohort analysis of players from 1999 to 2018. Orthop J Sports Med. 2021;9(9):23259671211024199. 35146029 10.1177/23259671211024199 PMC8822021

[6] Gotlin MJ , Minhas SV , Buchalter DB , Feder OI , Alaia MJ , Jazrawi LM . Performance and return to sport after hand, wrist, and forearm fractures in the National Hockey League. Arthrosc Sports Med Rehabil. 2020;2(5):e505–e510. 33134987 10.1016/j.asmr.2020.05.013 PMC7588639

[7] Hayes E , Meulenkamp B , Matache B , Pickell M . Achilles tendon ruptures in National Hockey League players: return to sport and performance impact. Foot Ankle Orthop. 2024;9(4):24730114241300153. 39619114 10.1177/24730114241300153 PMC11607746

[8] Hockey‐Reference.com. NHL stats, history, scores, standings, playoffs, schedule & records. In: Hockey‐Reference.com. Sports Reference LLC. Available via Hockey-Reference.com; 2025 [accessed 14 Dec 2025]. Available from: https://www.hockey-reference.com

[9] Hoenig JM , Heisey DM . The abuse of power: the pervasive fallacy of power calculations for data analysis. Am Stat. 2001;55(1):19–24.

[10] Jack RA , Sochacki KR , Gardner SS , McCulloch PC , Lintner DM , Cosculluela PE , et al. Performance and return to sport after Achilles tendon repair in National Football League Players. Foot Ankle Int. 2017;38(10):1092–1099. 28742993 10.1177/1071100717718131

[11] Khalil LS , Jildeh TR , Tramer JS , Abbas MJ , Hessburg L , Mehran N , et al. Effect of Achilles tendon rupture on player performance and longevity in National Basketball Association players. Orthop J Sports Med. 2020;8(11):2325967120966041. 33294475 10.1177/2325967120966041 PMC7708715

[12] Lantto I , Heikkinen J , Flinkkila T , Ohtonen P , Siira P , Laine V , et al. A prospective randomized trial comparing surgical and nonsurgical treatments of acute Achilles tendon ruptures. Am J Sports Med. 2016;44(9):2406–2414. 27307495 10.1177/0363546516651060

[13] LaPrade CM , Chona DV , Cinque ME , Freehill MT , McAdams TR , Abrams GD , et al. Return‐to‐play and performance after operative treatment of Achilles tendon rupture in elite male athletes: a scoping review. Br J Sports Med. 2022;56(9):515–520. 35144918 10.1136/bjsports-2021-104835

[14] Lemme NJ , Li NY , Kleiner JE , Tan S , DeFroda SF , Owens BD . Epidemiology and video analysis of Achilles tendon ruptures in the National Basketball Association. Am J Sports Med. 2019;47(10):2360–2366. 31268773 10.1177/0363546519858609

[15] Longstaffe R , Leiter J , MacDonald P . Anterior cruciate ligament injuries in the National Hockey League: epidemiology and performance impact. Clin J Sport Med. 2020;30(3):224–230. 32341289 10.1097/JSM.0000000000000584

[16] Mansfield K , Dopke K , Koroneos Z , Bonaddio V , Adeyemo A , Aynardi M . Achilles tendon ruptures and repair in athletes—a review of sports‐related Achilles injuries and return to play. Curr Rev Musculoskelet Med. 2022;15(5):353–361. 35804260 10.1007/s12178-022-09774-3 PMC9463425

[17] NHL Injury Viz. NHL Injury Viz: Index. In: NHL Injury Viz. Available via NHL Injury Viz; 2025 [accessed 14 Dec 2025]. Available from: https://nhlinjuryviz.blogspot.com/p/index-page.html

[18] O'Brien M . The anatomy of the Achilles tendon. Foot Ankle Clin. 2005;10(2):225–238. 15922915 10.1016/j.fcl.2005.01.011

[19] Ochen Y , Beks RB , van Heijl M , Hietbrink F , Leenen LPH , van der Velde D , et al. Operative treatment versus nonoperative treatment of Achilles tendon ruptures: systematic review and meta‐analysis. BMJ. 2019;364:k5120. 30617123 10.1136/bmj.k5120 PMC6322065

[20] Pearsall DJ, Turcotte RA, Lefebvre RL, Bateni H, Nicolau M, Montgomery D, et al. Kinematics of the foot and ankle in forward ice hockey skating. In: Proceedings of the 19th International Symposium for Biomechanics in Sports. University of San Francisco; 2001. p. 78–81.

[21] Schroeder GD , McCarthy KJ , Micev AJ , Terry MA , Hsu WK . Performance‐based outcomes after nonoperative treatment, discectomy, and/or fusion for a lumbar disc herniation in National Hockey League athletes. Am J Sports Med. 2013;41(11):2604–2608. 23956134 10.1177/0363546513499229

[22] Sim FH , Simonet WT , Melton LJ , Lehn TA . Ice hockey injuries. Am J Sports Med. 1987;15(1):30–40. 3812859 10.1177/036354658701500105

[23] Slawaska‐Eng D , Bouchard MD , Del Sordo L , Weber AE , Ayeni O . Performance and return to sport outcomes following hip arthroscopy in National Hockey League players. Knee Surg Sports Traumatol Arthrosc. 2025;33:2994–3001. 40501178 10.1002/ksa.12720 PMC12310084

[24] Sochacki KR , Jack RA , Hirase T , Vickery J , Harris JD . Performance and return to sport after hip arthroscopy for femoracetabular impingement syndrome in National Hockey League players. J Hip Preserv Surg. 2019;15(1):S–86–S–96.

[25] Swindell HW , McCormick KL , Tedesco LJ , Herndon CL , Ahmad CS , Levine WN , et al. Shoulder instability, performance, and return to play in National Hockey League players. JSES Int. 2020;4(4):786–791. 33345216 10.1016/j.jseint.2020.08.008 PMC7738589

[26] Tedesco LJ , Swindell HW , Anderson FL , Jang E , Wong TT , Kazam JK , et al. Evaluation and management of hand, wrist and elbow injuries in ice hockey. Open Access J Sports Med. 2020;11:93–103. 32425621 10.2147/OAJSM.S246414 PMC7196194

[27] Trofa DP , Miller JC , Jang ES , Woode DR , Greisberg JK , Vosseller JT . Professional athletes' return to play and performance after operative repair of an Achilles tendon rupture. Am J Sports Med. 2017;45(12):2864–2871. 28644678 10.1177/0363546517713001

[28] Trofa DP , Noback PC , Caldwell J‐ME , Miller JC , Greisberg JK , Ahmad CS , et al. Professional soccer players' return to play and performance after operative repair of Achilles tendon rupture. Orthop J Sports Med. 2018;6(11):2325967118810772. 30534574 10.1177/2325967118810772 PMC6280612

[29] Yang J , Hodax JD , Machan JT , Krill MK , Lemme NJ , Durand WM , et al. Factors affecting return to play after primary Achilles tendon tear: a cohort of NFL players. Orthop J Sports Med. 2019;7(3):2325967119830139. 30886876 10.1177/2325967119830139 PMC6415485

[30] Zhou K , Song L , Zhang P , Wang C , Wang W . Surgical versus non‐surgical methods for acute Achilles tendon rupture: a meta‐analysis of randomized controlled trials. J Foot Ankle Surg. 2018;57(6):1191–1199. 30368430 10.1053/j.jfas.2018.05.007

